# The topological soliton in Peierls semimetal Sb

**DOI:** 10.1038/s41598-024-52411-x

**Published:** 2024-01-28

**Authors:** Sergey V. Chekmazov, Andrei S. Ksenz, Andrei M. Ionov, Andrey A. Mazilkin, Anton A. Smirnov, Elena A. Pershina, Ivan A. Ryzhkin, Oleg Yu. Vilkov, Brian Walls, Kuanysh Zhussupbekov, Igor V. Shvets, Sergey I. Bozhko

**Affiliations:** 1 Institute of Solid State Physics, RAS, Chernogolovka, Russia; 2 Saint Petersburg State University, Saint Petersburg, Russia; 3 School of Physics, Centre for Research on Adaptive Nanostructures and Nanodevices (CRANN), Trinity College Dublin, Dublin, Ireland

**Keywords:** Topological defects, Electronic properties and materials, Surfaces, interfaces and thin films, Topological matter

## Abstract

Sb is a three-dimensional Peierls insulator. The Peierls instability gives rise to doubling of the translational period along the [111] direction and alternating van der Waals and covalent bonding between (111) atomic planes. At the (111) surface of Sb, the Peierls condition is violated, which in theory can give rise to properties differing from the bulk. The atomic and electronic structure of the (111) surface of Sb have been simulated by density functional theory calculations. We have considered the two possible (111) surfaces, containing van der Waals dangling bonds or containing covalent dangling bonds. In the models, the surfaces are infinite and the structure is defect free. Structural optimization of the model containing covalent dangling bonds results in strong deformation, which is well described by a topological soliton within the Su–Schrieffer–Heeger model centered about 25 Å below the surface. The electronic states associated with the soliton see an increase in the density of states (DOS) at the Fermi level by around an order of magnitude at the soliton center. Scanning tunneling microscopy and spectroscopy (STM/STS) measurements reveal two distinct surface regions, indicating that there are different surface regions cleaving van der Waals and covalent bonds. The DFT is in good agreement with the STM/STS experiments.

## Introduction

Su, Schrieffer and Heeger (SSH) developed a theoretical model to explain solitons in quasi-1D polyacetylene^1–3^. The Peierls instability leads to dimerization in polyacetylene and an alteration between single and double carbon bonds along the length of the polymer. The polymer is not symmetric to reflection through a mirror plane perpendicular to the 1D polymer length. However, a defect centered at the reflection plane can result in the defect-containing polymer being symmetric upon reflection through the plane. This defect violates the Peierls translational symmetry and is known as a topological soliton (TS). Experiments indicate that the TS defect extends over approximately 14 carbon atoms and can dramatically change the transport properties of the polymer^2,4^. Superconductivity of the polymer correlated to the presence of soliton has been predicted^5^. Therefore, the defect structure of Peierls insulators is of great interest.

Peierls predicted that the Peierls instability occurs in crystals of 3D topological semimetal Sb and Bi. Their crystal structure can be obtained from a simple cubic lattice by displacing every second (111) atomic plane in the [111] direction combined with altering the angle between the [111] vector and the other two <111> vectors by a few degrees^6^. Doubling of the translational period along the [111] direction in Bi and Sb results in bilayers wherein the two (111) planes of the bilayer are covalently bonded, while consecutive bilayers are bonded via van der Waals. The energy gain of the Peierls transition is a consequence of covalent bond creation^7^. The surface represents a violation of the Peierls condition. Due to the coexistence of covalent and van der Waals bonds, there can exist two different (111) surfaces of Sb containing covalent or van der Waals dangling bonds. Molotkov and Ryzhkin’s tight-binding approximation calculations predict that the two Sb(111) surfaces will exhibit distinct band structures^8^.

It is important to note that the surface with dangling covalent bonds may not exist due energy considerations, as cleaving van der Waals bonds is well established to be favourable^7^. Furthermore, the possibility and implications of structural relaxation of the surface containing covalent dangling bonds upon cleavage, has not been examined, to the best of our knowledge. In this work, we present the results of density functional theory (DFT) calculations of the Sb(111) surfaces. DFT simulations examining (111) terminated Sb with dangling covalent bonds at the surface reveal a strong deformation of the crystal lattice very similar to a TS in the (CH)_*x*_ chain. In addition to the soliton, this relaxation removes the dangling covalent bonds at the surface in favor of dangling van der Waals bonds. The results of the DFT simulations are supported by several experimental techniques including scanning tunneling microscopy (STM) and spectroscopy (STS), low-energy electron diffraction (LEED), angle-resolved photoemission spectroscopy (ARPES) with an ultraviolet source, X-ray photoelectron spectroscopy (XPS) and high-resolution transmission electron microscopy (HRTEM).

## Results and discussion

### Density functional theory calculations of crystal and electronic structure of Sb

**Figure 1:**
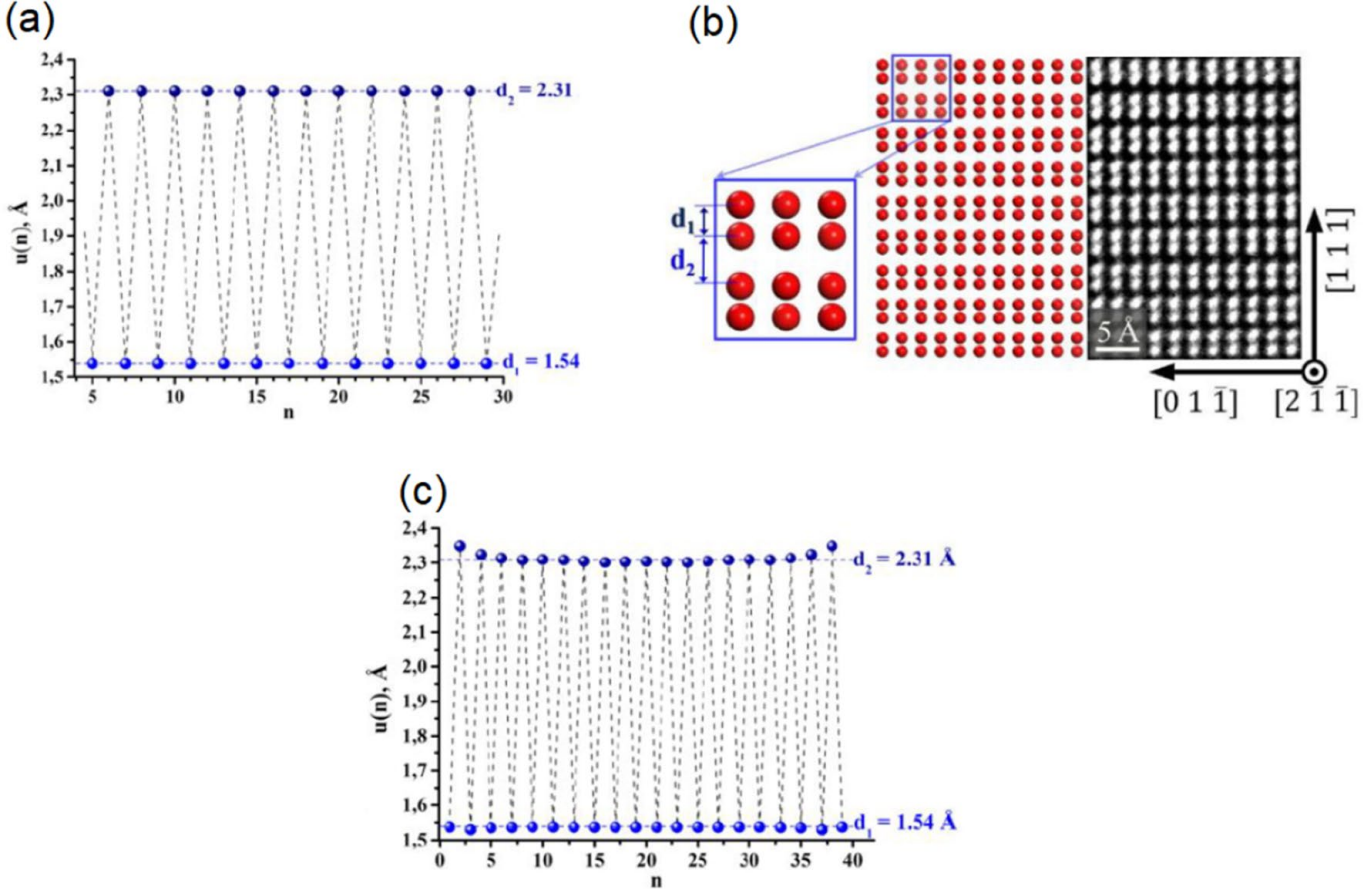
DFT calculation of bulk Sb and the (111) surface of Sb: **(a)** The interplanar distance along the [111] direction for bulk Sb. The interplanar distance alternates between 2.31 and 1.54 Å. **(b)** Left panel: Sb(111) structure after the DFT geometry optimization; right panel: high-resolution transmission electron microscopy image of Sb(111). **(c)** The interplanar distance along the [111] direction for (111) terminated Sb. The model is terminated by a bilayer and dangling van der Waals bonds. At the two surfaces (far left and right) the interplanar distance deviate slightly from the bulk-like interplanar distances, which is highlighted by the horizontal dashed blue lines. Away from the surfaces, the bulk-like interplanar distances are observed.

Prior to considering the (111) surface of Sb, the bulk structure is examined. Plane wave DFT calculations have been performed using the Vienna Ab initio Simulation Package (VASP) package^9–11^. Projected augmented-wave potentials^12^ were implemented for the ion–electron interactions. The generalized gradient approximation using the parametrization scheme of Perdew, Burke, and Ernzerhof^13,14^ was employed to treat the electron exchange and correlation. A plane wave cut-off of 400 eV was employed. The Brouillon zone was sampled by a 21 × 21 × 21 Monkhorst–Pack k-point sampling grid. Both the k-point grid and the cutoff energy were subjected to convergence testing. Structural optimization resulted in a rhombohedral structure with lattice parameters *a* = 4.594 Å and *α* = 56.96°, in agreement with the well-established structure^15–17^ and previous ab initio calculations^18–21^. Along the [111] direction the interplanar distance alternates between *d*_1_ = 1.54 Å and *d*_2_ = 2.31 Å, corresponding to covalent and van der Waals bonding, respectively^16,19,20,22,23^, and is depicted in Fig. 1a. The relaxed bulk structure is in good agreement with the HRTEM presented in Fig. 1b. Considering the spin–orbit interaction (SOI) led to a deviation of the lattice parameters by less than 0.5%. This structure is well described in terms of the SSH model^1–3^ whereby the interplanar distance can be expressed as a function of the (111) plane number *n*:

1 $$u\left(n\right)= {\left(-1\right)}^{n}0.385+1.925,$$

where *u*(*n*) is the atomic interplanar separation in the [111] direction between the *n*_*th*_ and *n*_*th*_ + 1 layers. 1.925 is the average interplanar distance in the [111] direction. 0.385 is equal to the *d*_2_ minus 1.925.

In the following calculations considering the [111] surface, the same cut-off energy and functionals are employed and the SOI is implemented. The k-point sampling is reduced in the [111] direction due to the larger real-space size of the model. The repeated-slab geometry^24^ is employed to model Sb(111). A vacuum gap of 20 Å separates the slabs. The model contains two surfaces on either side of the vacuum gap. Sb and similar materials such as Bi are often discussed in terms of bilayers^19–22,25^. A bilayer consists of two (111) planes covalently bonded. Consecutive bilayers are bonded via van der Waals. Slabs with [111] surfaces can be terminated by either a bilayer and van der Waals dangling bonds, or by a (111) plane not in a bilayer and covalent dangling bonds. The former is energetically favorable due to cleavage of the weaker van der Waals bond. We will consider both of these surface terminations. Throughout the text, we refer to the two surface terminations as the van der Waals (vdW) and covalent (CV) surfaces, reflecting the dangling bonds at the surface of the models prior to structural optimization.

We first present the calculation of the vdW surface. The model, referred to as Sb40, consists of 40 (111) layers, or 20 bilayers, and is sampled by a 21 × 21 × 1 Monkhorst–Pack k-point sampling grid. The geometry optimization is presented in Fig. 1c. The interplanar distance, *u*(*n*), alternates between 2.31 and 1.54 Å illustrated by the horizontal dashed lines in Fig. 1c. These interplanar distances agree with the bulk calculation in Fig. 1a. The only deviation from the bulk-like interplanar distances is observed at the two surfaces at the right and left of Fig. 1c, where a small (less than 2%) relaxation of the interplanar distance is observed in accordance with^22^, while the lateral distortion of the surface structure does not exceed 0.5%.

**Figure 2:**
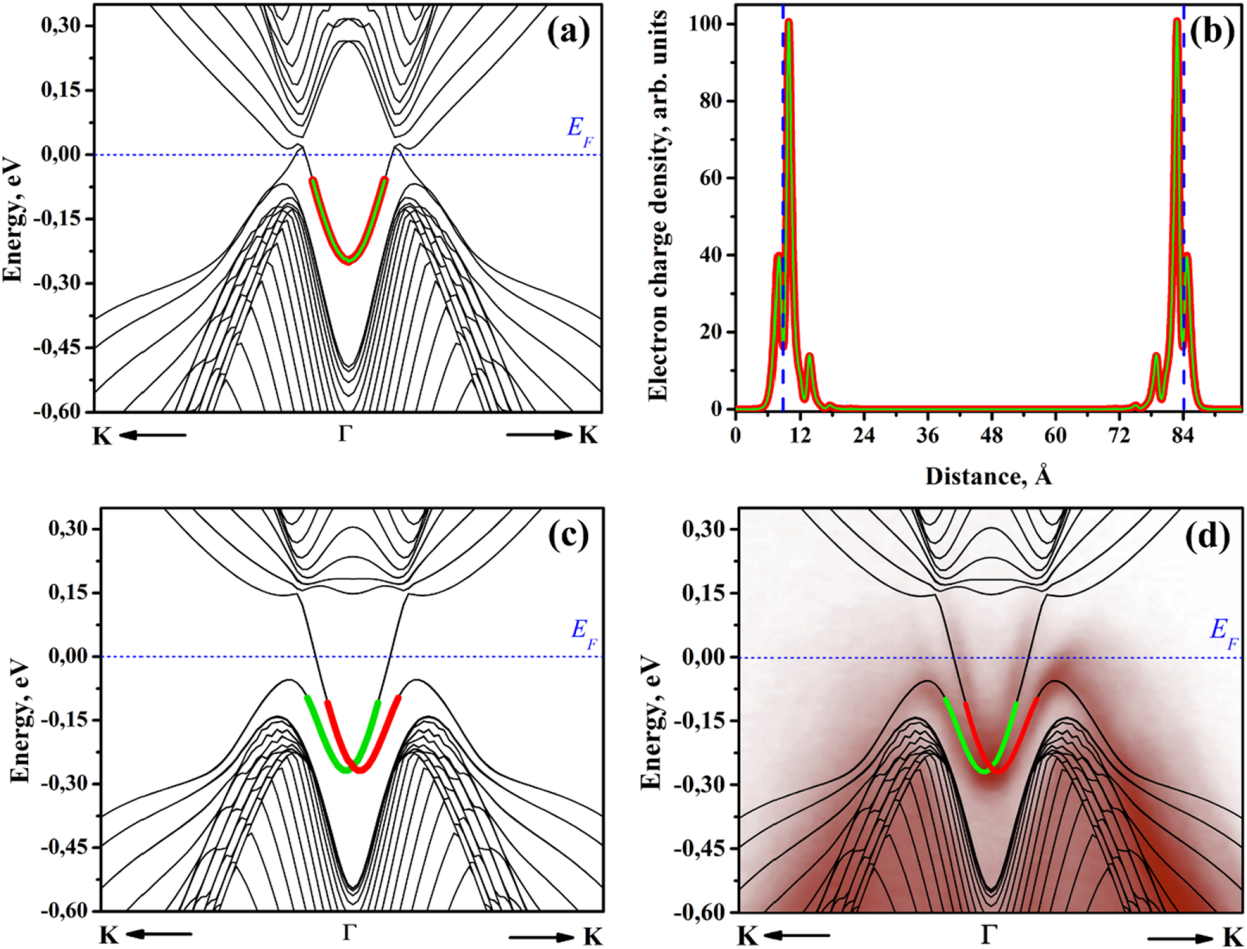
(**a**) DFT calculation of the electron dispersion in the *К*–*Г*–*К* direction of Sb40 without considering SOI. (**b**) The spatial charge density of the green and red bands in (**a**) de-convoluted into the individual (111) planes. The dashed blue vertical lines highlight the two surfaces of the Sb40 model. The red and green bands show strong surface character. (**c**) DFT calculation of the electron dispersion in the *К*–*Г*–*К* direction of Sb40 including the SOI. (**d**) ARPES (red half tone) and the DFT simulations of the electron dispersion including the SOI are in good agreement.

The electron dispersion of Sb40 has been calculated without and with consideration of the SOI and is depicted in Fig. 2a,c. The red and green correspond to the surface states (SS) of the two spin channels. The electron charge density of the red and green band is plotted as a function of distance from the surface in Fig. 2b. The vertical dashed blue lines indicate the two surfaces of Sb40. The charge density in these regions highlight that the bands are located at the surface. The bottom of the surface state branch at the *Г* point is ${\varepsilon }_{1}=0.25\text{ eV}$ below the Fermi level. The spin degeneracy of the surface state is lifted by the SOI via splitting in the k direction (Fig. 2c). Bulk Sb behaves as a topological insulator, leading to the emergence of helical Dirac states due to the bulk-boundary correspondence^26–28^. The electron dispersion has been measured by ARPES and is overlayed as the red halftone in Fig. 2d. A massless Dirac cone at the *Г* point with a Dirac point ${\varepsilon }_{DP}=0.26\text{ eV}$ below the Fermi level was measured by ARPES in agreement with the simulation. Note that in the vicinity of the surface the SOI is determined by the pseudopotential gradient *∂U/*∂Z^29^*,* which has opposite sign at each side of the Sb40 model. Therefore, the red and green bands, corresponding to the two spin channels will be reserved at each side of the symmetric Sb40 model.

**Figure 3:**
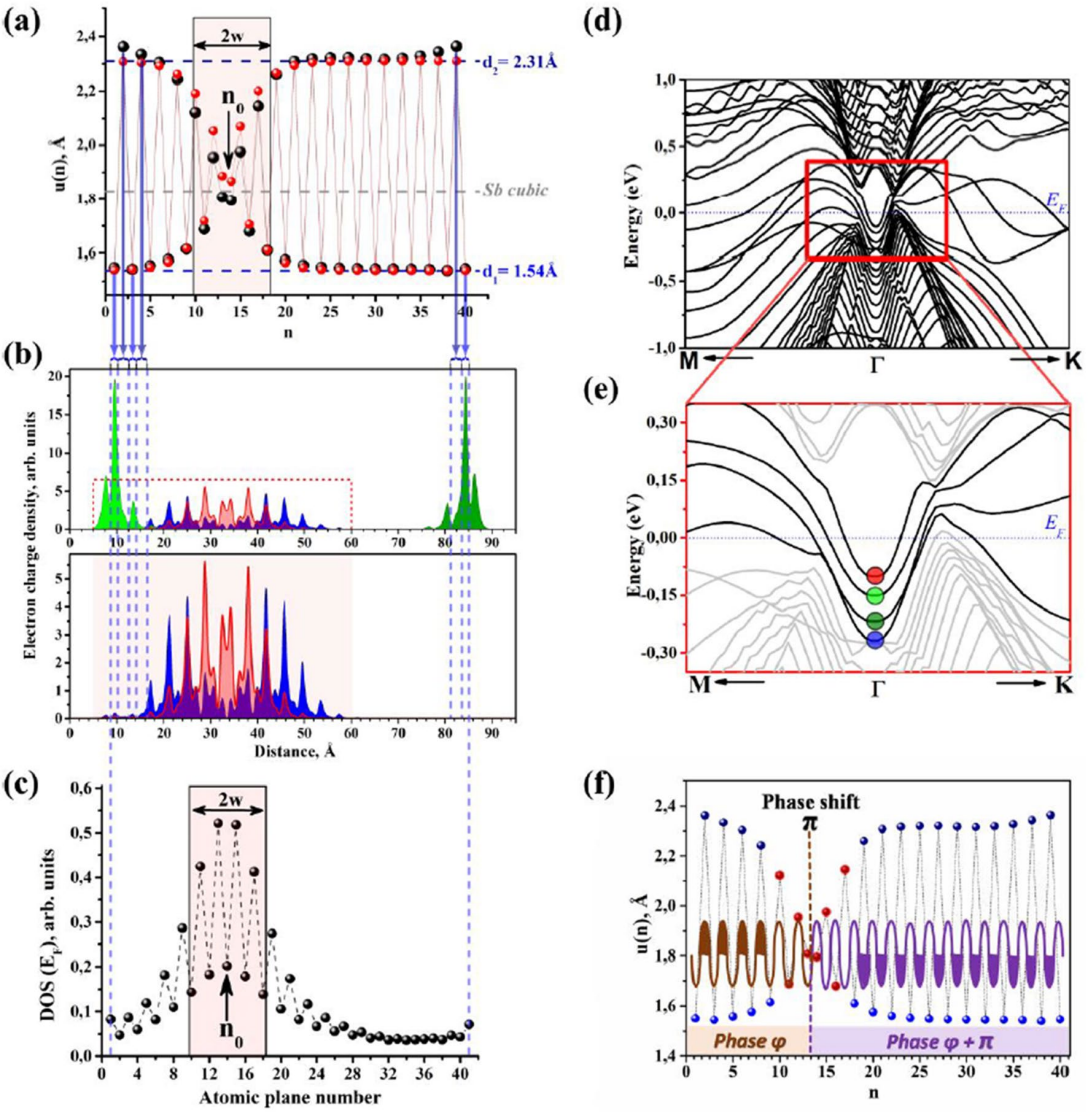
DFT simulation of the Sb41 model, which consists of 41 (111) planes. In the initial model, one of the surfaces is terminated by a bilayer while the other surface is terminated by a single plane with dangling covalent bonds. **(a)** The interplanar distances along the [111] direction. The dashed blue horizontal lines highlight the bulk-like interplanar distances, while the dashed gray horizontal line highlights the interplanar distance of the cubic phase of Sb. The black circles indicate the DFT calculation while the red circles indicate the fit from Eq. (2), which describes a TS in the SSH model. The two-dimensional defect at the surface, in the form of a (111) plane with dangling covalent bonds, results in a considerable structural distortion. **(b)** Spatial charge density distribution of the electron dispersion branches (marked in color) of electrons at the *Г* point of the Brillouin zone. **(c)** Spatial distribution of the Fermi level DOS averaged over (111) planes. The planes are aligned with (**a,b**). The soliton region sees an order of magnitude increase in the DOS at the Fermi level. The black arrow indicates the position of the TS center, *n*_0_, while the rectangle indicates the TS size, *w,* obtained from Eq. (2). **(d)** DFT calculation of the electron dispersion in the M–Г–К direction without considering the SOI. **(e)** Zoom of the red square in (**d**). Four dispersion bands are highlighted by red, blue, dark green and light green. Their spatial distribution within the slab can be visualized in (**b**). The red and blue bands are TS soliton states while the two green bands are surface states at either side of the slab. Note the non-equivalence of the two surfaces due to the proximity of one of the surfaces to the TS. **(f)** The interplanar distances along the [111] direction. The sinusoid represents the alternating interplanar distance of bulk Sb. The half-filled part indicates the van der Waals distance of 2.31 Å. As one passes through the TS region, the interplanar distance undergoes a phase change of π.

Calculations considering the CV surface consist of an odd number of (111) atomic layers. Models containing 9, 21, 31, 41and 51 layers are examined. The geometry optimization of Sb41 is presented in Fig. 3a. Despite the model being terminated by a single (111) layer not part of a bilayer, the geometry optimization results in a bilayer terminating the surface. Therefore, the structural relaxation removes the dangling covalent bonds in favor of van der Waals dangling bonds. Below this “regular” surface there is a strong structural distortion. A defect region of 8–10 interplanar distances can be visualized in Fig. 3a. The interplanar separation in this region deviates from the bulk distance, which alternates between 2.31 and 1.54 Å and is highlighted by the blue dashed horizontal lines in Fig. 3a). In the center of this defect region the interplanar distance is 1.83 Å (grey dashed horizontal line in Fig. 3a), which is very close to the lattice parameter of the metallic cubic phase of Sb^7,30–32^. We note that the Peierls-like alteration of the interplanar distance is diminished in the defect region, but persists elsewhere. One can consider the structural relaxation as the diffusion and considerable modification of a two-dimensional defect. In the initial model, the single (111) layer with dangling covalent bonds is the two-dimensional defect. Upon relaxation, the defect diffuses away from the surface where it forms the stable soliton.

Strong deformation of the rhombohedral Sb structure has been observed where the material is subjected to high pressure. Phase transitions to SbII and SbIII were realized under 8–12 GPa^17,18,33–35^ and 28 GPa^17,36^, respectively. In addition, an incommensurate composite phase, termed SbIV, with a monoclinic structure was observed at pressure between the aforementioned pressures. However, the metallic simple cubic phase was also observed^37–41^.

The interlayer distances in the defect region of Sb41 can be described as a TS in the SSH model^1–3^:

2 $$u\left(n\right)= {\left(-1\right)}^{\left(n-1\right)}0.385{\text{tanh}}\left(\frac{n-{n}_{0}}{w}\right)+1.925,$$

where *n*_0_ is the center of the TS and *2w* is the TS size, which were both determined by fitting with respect to the DFT calculation. In Fig. 3a the structure of the defect region simulated by DFT (black circles) and calculated by Eq. (2) (red circle) are compared. In Fig. 3f the oscillation of the atomic interplanar distance is illustrated by the schematic sinusoid. The sinusoid has a period of 3.85 Å corresponding to the translational period of rhombohedral Sb along [111]. This corresponds to the sum of the van der Waals and Covalent interplanar spacings. The half-filled part of the sinusoid indicates the location of the van der Walls spacing. As one passes through the soliton region, the van der Waals spacing undergoes a phase change of π. This behavior has been observed previously in a Coulomb crystal^42^.

The deviation at the surface is due to surface relaxation which the SSH model does not account for. The TS is located 25 Å below the surface and its size is 16.4 Å. For CV models consisting of 9 and 21 (111) layers the soliton is located in the middle of the slab. This clearly occurs as the slab is not large enough to adequately describe the TS. The position of the soliton in Sb31, Sb41 and Sb51 illustrates it does not diffuse into the bulk but is stable near the surface. The displacement of the soliton from the terminating layer is an important point to be taken into account when planning experiments to visualize the soliton.

In order to identify the branches of the electron spectra that correspond to the soliton, DFT calculation of the electron dispersion were performed without considering the SOI, as this facilitates visualizing the spatial charge density distribution at the *Г* point without the spin–orbit induced splitting. Figure 3d demonstrates the simulated electron dispersion of the Sb41 structure. The simulated electron dispersion around the *Г* point is visualized in Fig. 3e, corresponding the red box in Fig. 3d. The 4 bands closest to the Fermi level at the *Г* point are marked by the four circles. The electron charge density at the *Г* point of these four bands, as a function of the distance from the surface, is plotted in Fig. 3b. The blue and red bands are distributed in the soliton region, while light and dark green bands are located the surfaces close and far from the soliton, respectively. The surface band furthest from the soliton (separated from the TS by 52.9 Å) in dark green is located ${\varepsilon }_{SS1}=0.218\text{ eV}$ below the Fermi level at the *Г* point. The surface band closest to the soliton in light green is ${\varepsilon }_{SS2}=0.15\text{ eV}$ below the Fermi level at the *Г* point. The bands of the soliton are located ${\varepsilon }_{TS2}=0.1\text{ eV}$ (red) and ${\varepsilon }_{TS1}=0.27\text{ eV}$ (blue) below the Fermi level at the *Г* point.

**Figure 4:**
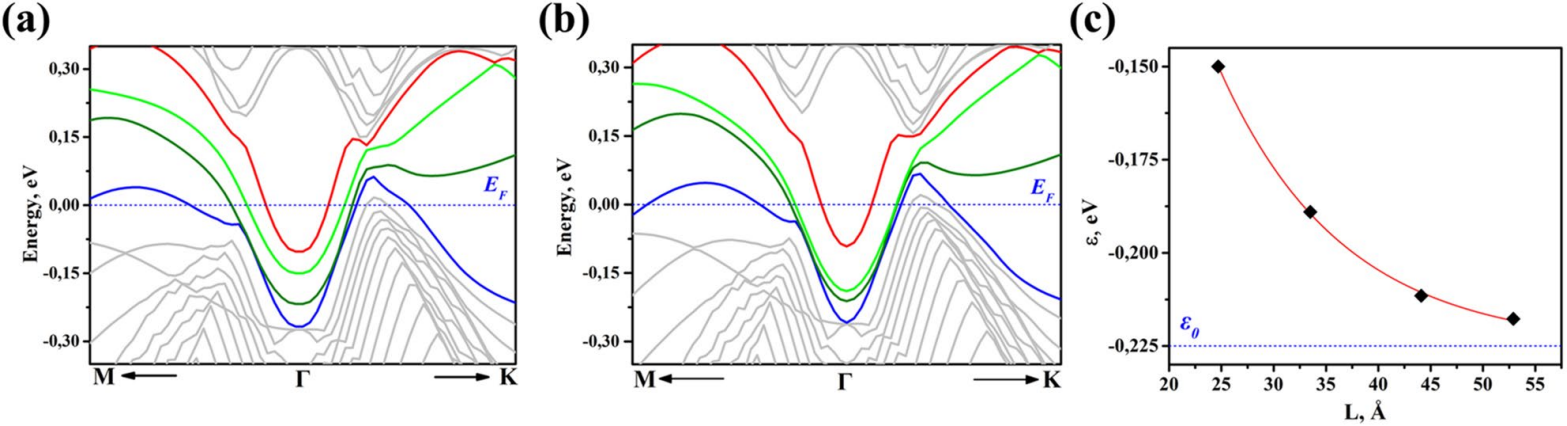
DFT simulated electron dispersion in the M–Г–К direction without considering the SOI. Models contain 41 atomic layers in which (**a**) the soliton is 25 Å below the surface and (**b**) 33.5 Å below the surface. The red and blue bands are TS states, while the light and dark green bands are SS bands close and far from the soliton, respectively. When the soliton is moved away from the surface towards the center of the slab the light green SS band is modified. (**c**) Dependence of the position of the SS bands at the *Г* point on the SS-TS separation. The data points corresponds to the calculated data, light and dark green in (**a,b**), while the red curve represents the fit of the calculated data by an exponential decay $$\varepsilon =-0.225+0.614{\text{exp}}(-$$ *L*/11.8$$)$$.

To clarify the role of the soliton’s interaction with the surface electrons, DFT simulations of 41 layers model (termed Sb41*) with the soliton located 33.5 Å below the surface have been performed. In the case of Sb41, which contains a monolayer at the surface prior to structural optimization, the soliton resides 25 Å below the surface after the structural optimization. Electron dispersion of the Sb41 and Sb41* structures are presented in Fig. 4a,b. The bands of the TS in Sb41* are located $${\varepsilon }_{TS2}^{*}=0.09\text{ eV}$$ (red) and $${\varepsilon }_{TS1}^{*}=0.26\text{ eV}$$ (blue) below the Fermi level at the *Г* point. Both of the TS bands are not substantially affected by the TS location—$${\varepsilon }_{TS1}^{*}$$ and $${\varepsilon }_{TS2}^{*}$$ are very close to $${\varepsilon }_{TS1}$$ and $${\varepsilon }_{TS2}$$, respectively. The SS furthest from the TS (separated from TS by 44.1 Å) in dark green and the SS closest to the TS in light green are located at $${\varepsilon }_{SS1}^{*}=0.211\text{ eV}$$ and $${\varepsilon }_{SS2}^{*}=0.189\text{ eV}$$ below the Fermi level at the *Г* point. The dependence of the energy of the SS on the SS-TS separation, *L,* based on simulations of Sb41 and Sb41* is presented in Fig. 4c. The calculated values of *ε* is well fitted by an exponential decay $$\varepsilon =-{\varepsilon }_{0}+A{\text{exp}}(-$$*L*/λ$$)$$, where $${\varepsilon }_{0}=0.225\text{ eV}$$ $$A=0.614\text{ eV}$$ and λ = 11.8 Å. Note, $${\varepsilon }_{0}$$ is close to $${\varepsilon }_{1},$$ the energy of the SS in Sb40, which does not contain any soliton. λ is determined by the overlap of the SS and TS electron wave functions. λ is comparable with soliton size 16.4 Å since the SS wave function decays much faster than that of the TS. The energy of the SS as a function of the position of the TS relative to the surface reveals that the interaction between the SS and the TS dictates the positon of the soliton. The soliton becomes stable 25 Å below the surface. At large SS-TS distances the SS-TS interaction is negligible and soliton can be located at any position.

The lattice deformation and formation of the soliton results in the modification of the DOS. The DOS averaged over each (111) atomic plane is plotted in Fig. 3c. An increase in the DOS by nearly an order of magnitude is observed at the center of the soliton in comparison to the bulk-like region on the right side of the figure. We also note in this region the DOS oscillates from plane to plane. The CV and vdW surfaces are both terminated by a bilayer with vdW dangling bonds after the geometry optimization. However, in Fig. 3b one can see the different electron density of states in the presence of the soliton (light green) and at the vdW surface (dark green, far right). The difference is due to two factors: (i) the tail of an electron wavefunction in the soliton region and (ii) the aforementioned interaction of the soliton and surface electrons (overlapping of the soliton and surface wavefunctions is visible in Fig. 3b). The modification of the surface DOS in the presence of the soliton is crucial for the interpretation of STM and LEED discussed in “Experimental observation of topological solitons at the Sb(111) surface of the single crystal” section.

In order to understand if the soliton can be realized in an experiment we have estimated the formation energy for the different Sb(111) surfaces by DFT calculations (see Supplemental Material (SM)). We consider the vdW surface (E_vdW_) and the relaxed (*E*_S_) and unrelaxed CV (E_CV_) surface. To reiterate, the relaxed CV surface results in the soliton. *E*_vdW_ = 0.31 eV, *E*_CV_ = 1.575 eV and *E*_S_ = 0.78 eV. Dangling CV bonds are unstable in comparison the vdW dangling bonds. The relaxation of the CV surface giving rise to the soliton reduces the formation energy considerably, however the vdW surface is still the most favorable. Therefore, one can predict that the cleaved Sb(111) will predominantly be terminated by the vdW surface. It is conceivable that cleaving the surface revealing dangling CV bonds can lead to the surface relaxation resulting in the soliton state.

In reality, if the vdW and CV surface coexist the boundary between the two surface regions can influence the formation and position of the soliton. We observe that at the step edge between the two regions, the soliton is pinned to the surface. However, this is highly localized effect as the deformation move away from the surface further from the step edge defect. This is discussed in Sect. G of the SM.

### Experimental observation of topological solitons at the Sb(111) surface of the single crystal

To reduce the density of inhomogeneities high purity Sb single crystals were used in our experiments (see Sect. A of SM). Cleaving (111) terminated Sb should predominantly result in surfaces with dangling van der Waals bond due to the weak bond strength compared to the covalent bond. A number of STM experiments were performed on about 15 cleaved Sb(111) samples. In total, around 100 regions were investigated by STM. However, the CV surface region was only observed in 3 of our experiments.

**Figure 5:**
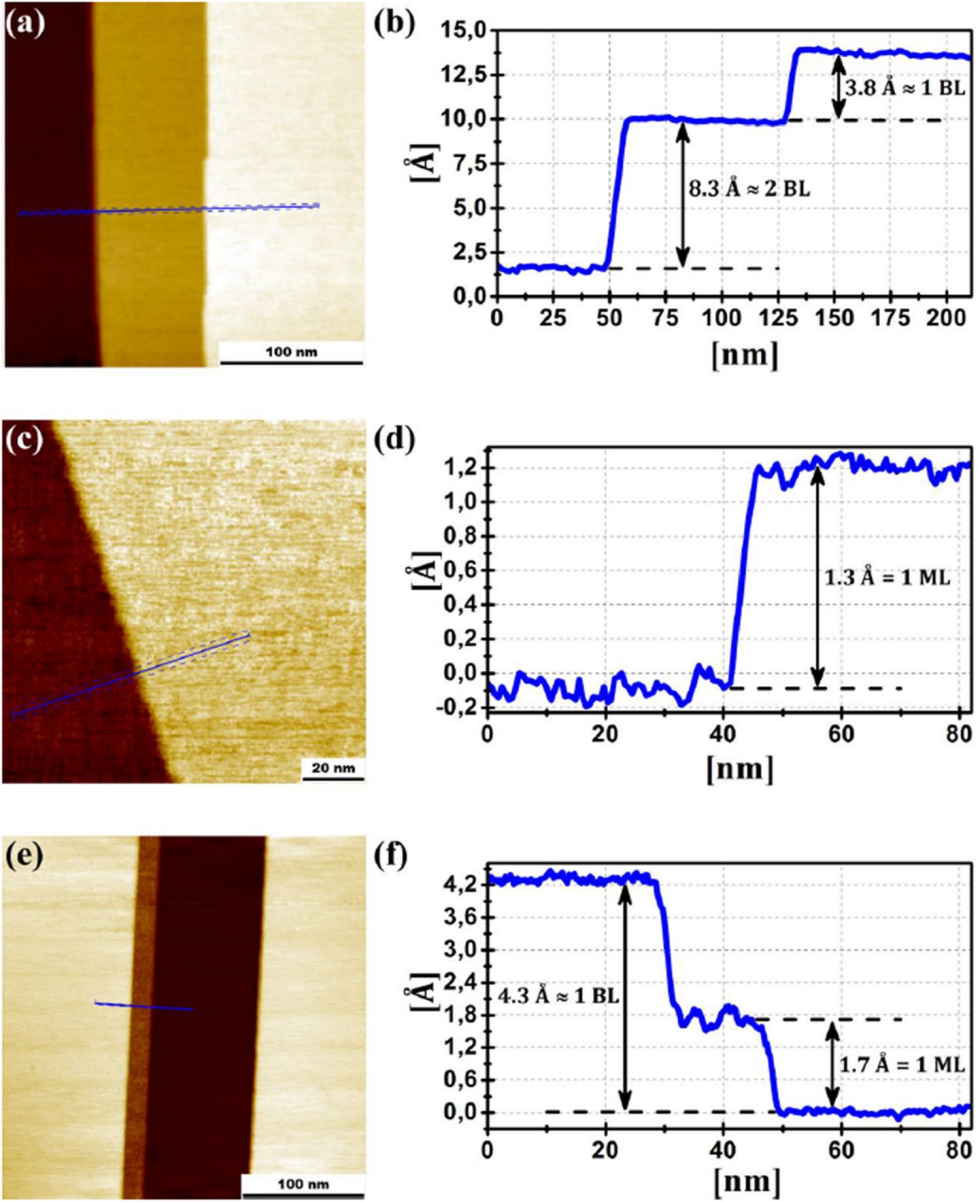
(**a**) 255 × 255 nm^2^ STM image of Sb(111), *U*_bias_ =  + 0.2 V, *I*_tun_ = 70 pA. (**b**) The corresponding cross-section of the STM image along the blue line in (**a**). (**c**) 120 × 120 nm^2^ STM image of the Sb(111), *U*_bias_ =  + 0.6 V, *I*_tun_ = 300 pA. (**d**) The corresponding cross-section of the STM image along the blue line in (**c**). (**e**) 300 × 300 nm^2^ STM image of the Sb(111) surface, *U*_bias_ =  + 0.3 V, *I*_tun_ = 100 pA. (**f**) The corresponding cross-section of the STM image along the blue line in (**e**). Bilayer (BL) and monolayer (ML) steps are indicated in the cross sections. All images were acquired at room temperature using the Pt-Ir tip.

In Fig. 5a–c STM images of cleaved Sb(111) is shown. Figure 5a consists of three terraces separated by sharp step edges. The step height *h*_*v*_, measured by STM, will predominantly be a multiple of the translational period in the [111] direction, equal to to 3.8 ± 0.1 Å. This corresponds to the distance between consecutive bilayers. The line profile in Fig. 5d,f corresponds to the line segment in Fig. 5c,e, respectively. We observe step heights considerably less that the translational period. This can only be understood as cleavage of van der Waals and Covalent bonds on either side of the step. Previously we have observed monolayer steps on Sb(111) and Bi(111) after ion bombardment of the cleaved surface (see SM and Refs.^43,44^). The monolayer step height measured by STM is extremely hard to estimate. The DFT calculations predict that cleaving covalent bonds results in significant relaxation along the [111] direction resulting in the soliton. From the DFT we can estimate the geometric monolayer step height as the difference in the thickness of the Sb40 and Sb41 models, giving a value of 1.8 Å. One should note the two terraces are connected at the step edge and along a two-dimensional plane into the surface, which in theory can modify the geometric monolayer step height. However, the greatest challenge in estimating the step height observed by STM is that STM probes the DOS of the surface. A different DOS due to a different surface will result in a different tip-surface distance in each case.

**Figure 6:**
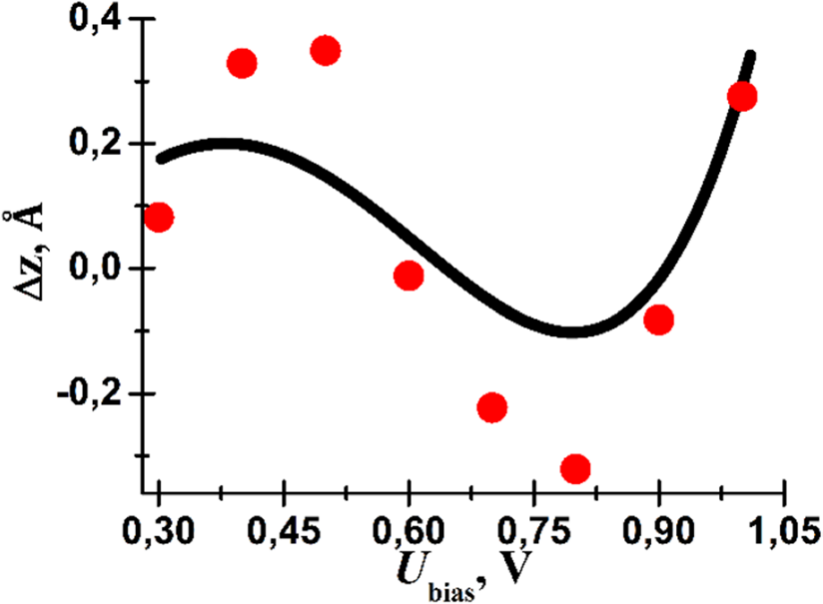
Dependence of the variation in monoatomic step height measured by STM, *Δz*, on the applied bias voltage. The red circles indicate experimental data. The black curve represents the fit of the experimental data obtained from Eq. (3). *k* in Eq. (3) is determined by the work function, electron energy and its effective mass. The presented best fit corresponds to an effective electron mass about 0.1m_e_ (effective mass of electrons in Sb in the [111] direction is about 0.1m_e_^45,46,47^). The STM experiment was performed at bias voltages where the difference in DOS on either side of the monoatomic step is significant according to the DFT simulations (Fig. 7a).

The monolayer step height measured by STM can be defined as: ${h}_{v}=H\pm \Delta z$, where “+” corresponds to the vdW → CV shift, “−” corresponds to the CV → vdW, and

3 $$\Delta z=\alpha {\text{ln}}\left(\frac{\underset{0}{\overset{e{U}_{{\text{bias}}}}{\int }}{\rho }_{{\text{tip}}}\left({E}_{F}-e{U}_{{\text{bias}}}+E\right){\rho }_{{\text{CV}}}\left({E}_{F}+E\right)dE}{\underset{0}{\overset{e{U}_{{\text{bias}}}}{\int }}{\rho }_{{\text{tip}}}\left({E}_{F}-e{U}_{{\text{bias}}}+E\right){\rho }_{{\text{vdW}}}\left({E}_{F}+E\right)dE}\right),$$

where *U*_bias_ is the sample bias with respect to the tip, *ρ*_tip,_ *ρ*_CV_ and *ρ*_vdW_ are the DOS of the STM tip, CV and vdW surfaces, respectively and *E*_*F*_ is the Fermi level. Estimation of *Δz* as a function of bias voltage from DFT calculations is presented as the black curve in Fig. 5. The real step height measured by STM in presented as the red points Fig. 6.

**Figure 7:**
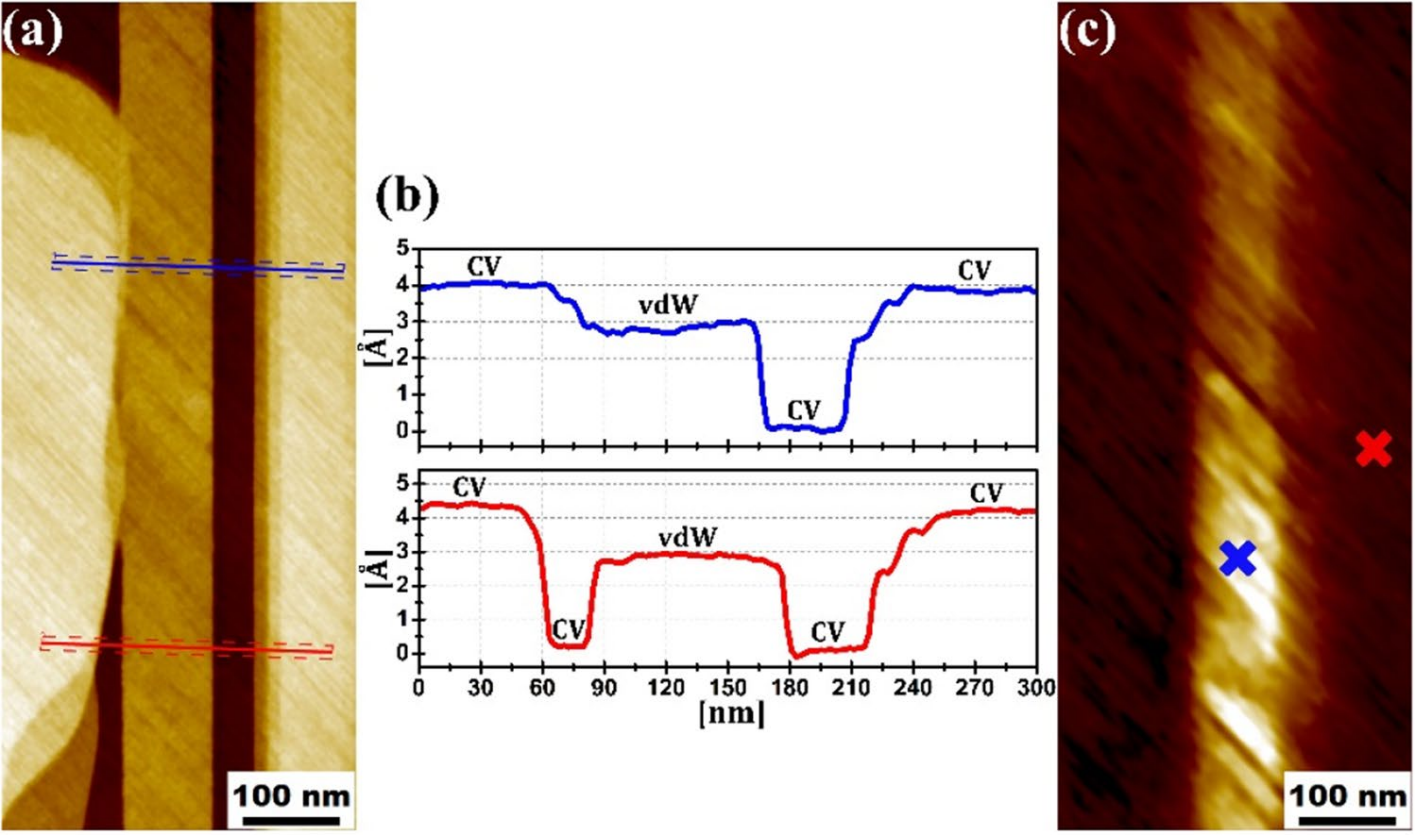
(**a**) Large scale (365 × 850 nm^2^) STM topography image. (**b**) Cross-sections of the STM image along the blue and red lines, respectively, in (**a**). “CV” and “vdW” indicate CV and vdW regions of the surface. (**c**) d*I*/d*V* map corresponding surface area in (**a**). *U*_bias_ =  + 0.2 V, *I*_tun_ = 300 pA. *U*_mod_ = 20 mV. We observe two distinct regions.

**Figure 8:**
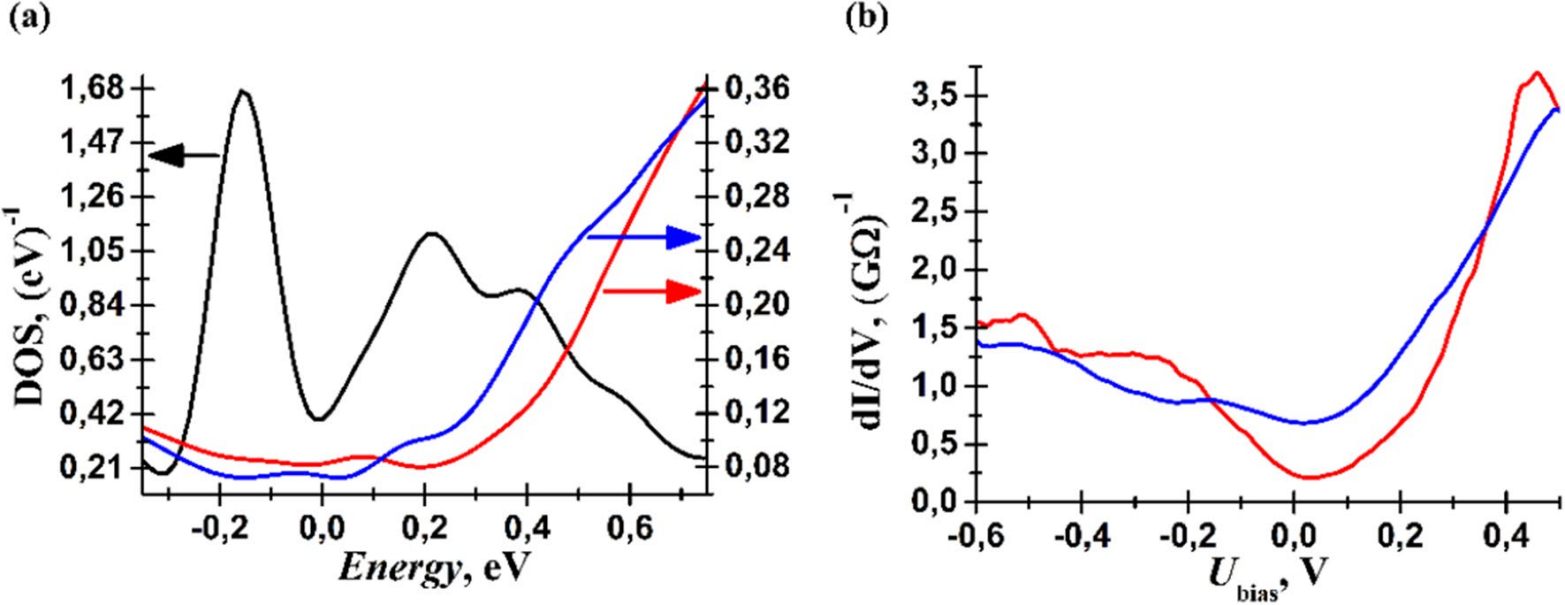
(**a**) Calculated DOS of the top surface layer of the Sb40 (blue curve) and Sb41 (red curve) models. The black curve corresponds to DOS of unrelaxed Sb41 (unreconstructed monolayer-terminated Sb(111) surface with dangling covalent bonds) (**b**) Tunneling spectra of two non-equivalent surface regions, obtained at the blue and red crosses in Fig. 6c.

The electronic properties of the two surfaces has been examined by STS measurements. Figure 7a,c are STM and d*I/*d*U* images obtained simultaneously on a surface area consisting of several terraces. The d*I/*d*U* map is insensitive to the tip height and probes the LDOS. We observe two regions electronically non-equivalent visualized as the dark and bright vertical areas in Fig. 7c. The blue and red line profiles in Fig. 7b, corresponding to the line segments in (a), reveal the steps heights. The step height of around 4 Å corresponds to the translational period, and hence, the terraces on either side of these steps are electronically equivalent. This is in agreement with the d*I/*d*U* map. On the other hand, the step height of around 3 Å is a monolayer step and terraces on either side of these steps should be electronically non-equivalent. Therefore, the vertical terrace through the center of (a)—which is 3 Å above its two neighbouring terraces—should be non-equivalent to the other areas of the surface. This is captured by the d*I/*d*U* map where we see a vertical band in the same region. In Fig. 8b we depict two point STS measurements performed on the two region in Fig. 7c highlighted by the blue and red crosses. In Fig. 8a we present the DFT calculated surface layer DOS that can be compared to the STS in Fig. 8b. Blue is the calculated top surface layer DOS of the relaxed Sb40 (vdW model of the Sb(111) surface); red is Sb41 (CV model containing soliton after geometry optimization); black is the unrelaxed Sb41 (unreconstructed monolayer-terminated Sb(111) surface with dangling covalent bonds). Considering the blue and red curves, we observe qualitative agreement. Dangling covalent bonds at the surface leads to an order of magnitude increase in the DOS with 3 prominent peaks at − 0.16 eV, 0.22 eV and 0.38 eV in comparison with bilayer terminated surfaces. Based on these set of spectroscopy measurements we tentatively suggest that the soliton is observed experimentally in the vicinity of the surface.

**Figure 9:**
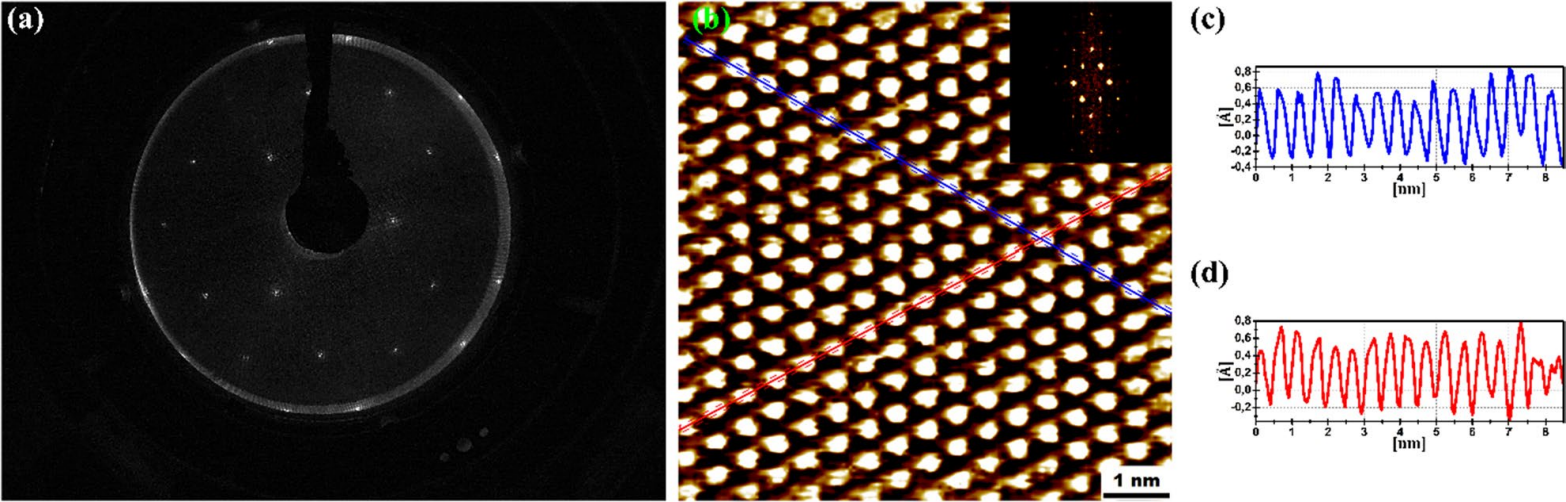
(**a**) LEED pattern of the cleaved Sb(111) surface. *E*_*p*_ = 112 eV. (**b**) STM image of 7.5 × 7.5 nm^2^ with atomic resolution measured with the Pt-Ir tip at *T* = 300 K, *U*_bias_ =  + 0.01 V, *I*_tun_ = 70 pA. Insert: The Fast Fourier transform of the STM image. (**c,d**) Blue and red profiles of corresponding cross-sections in the STM image in (**b**).

Surface reconstruction and relaxation occurs at the surface of crystalline materials due to the different coordination of the atoms compared to the bulk. Usually, cleaving covalent bonds at the surface result in the formation of new bonds, resulting in a surface reconstruction^48,49^. The atomic structure of the cleaved Sb(111) surface has been examined by STM and LEED. The diffraction pattern (Fig. 9a) is indicative of an unreconstructed 1 × 1 crystal. Note that the coverage of the surface where covalent bonds are cleaved is assumed to be small on the basis of the formation energy. Therefore, if a surface reconstruction occurs where covalent bonds are cleaved, it will likely not be observed by area averaging LEED. However, a separate study conducted by the authors examining ion bombarded Sb(111) surface is worth noting^43^. The ion-bombarded surface contains a large coverage of areas cleaved by covalent bonds. Nevertheless, no structural surface reconstruction was observed by LEED. The lack of a surface reconstruction can be understood by considering the DFT calculations. In the case of the CV surface, where the initial model contained covalent dangling bonds, structural optimisation resulted in a regular bilayer with dangling van der Waals bonds terminating the model. Such a termination will result in the regular 1 × 1 LEED pattern. Note that the electron mean free path at low energies characteristic of LEED is small. Therefore, the LEED technique is not suitable for probing crystal lattice deformation such as the soliton located 16.5–33 Å below the surface. STM provides a localized probe of the atomic structure. STM measurements, depicted in Fig. 9b have been performed on surface areas which exhibit modified electronic character as determined by STS measurements in Fig. 7b. However, the STM images are the same characterised by an interatomic distance of 5.0 ± 0.5 Å and a (1 × 1) hexagonal structure illustrated by the lines profiles in Fig. 9c,d.

### Discussion

The SSH theory was originally being developed for 1D structures. Here we discuss the soliton in 3D Sb crystal in terms of the SSH theory. This approach yields good agreement, revealing that the SSH model can be applied to 3D materials. One of the main consequences of the formation of the soliton is the drastic change in the electron structure, which can be considered a ground for the observation of superconductivity. Superconductivity in pure Sb has been observed at high pressure: at 2.6–2.7 K (120 kbar compressive stress and subsequent cooling)^50^, at 3.55 K (85 kbar compressive stress and subsequent cooling)^51^ and at 3.4 K (150 kbar compressive stress after cooling)^51^. Note, that in all case the superconductivity was observed under large compressive stress. Another argument in favour of possible superconductivity is the Peierls transition in Sb, which justifies strong electron–phonon interaction. Therefore, the predicted increase in the DOS in the soliton region where the crystal lattice undergoes significant deformation, leads us to speculate that the soliton may exhibit superconductivity.

The Peierls instability was originally conceptualized in a one-dimensional lattice with electron band half-filling that leads to spontaneous dimerization. Due to the nesting of the 1D Fermi surface, metallic states becomes unstable even in the presence of an infinitesimally weak interaction. This manifests itself in the logarithmic divergence of the electronic susceptibility at momentum q = 2k_F_^52^. In two or three dimensions, the nesting of the Fermi surface areas is less likely, and the metallic state is stable generally speaking. However, specific lattice and corresponding electronic spectrums can give rise to Fermi nesting favourable for the Peierls transition, i.e., the electron energy decrease exceeds the increase in the lattice energy upon dimerization. In such cases, even in 2D and 3D dimensions, a lattice distortion or a charge density wave occurs with a certain momentum q^6^. Our results demonstrate that soliton state in the SSH model can be realized not just in 1D structures like a polyacetylene and 1D chain of Rb atoms^53^, but also in 3D materials.

The possibility of two different (111) surfaces upon cleavage—containing covalent or van der Waals dangling bonds—means there can exist two distinct topological states where the Peierls conditions is violated. The DFT model containing dangling covalent bonds reconstructs at the surface resulting in the removal of the dangling covalent bonds in favour of dangling van der Waals bonds and the soliton. Therefore, the (111) terminated Sb is always terminated by a complete bilayer with dangling van der Waals bonds, regardless of how it was cleaved. The structural relaxation and electronic states associated with the soliton can influence electronic character of the surface. The electron dispersion of the soliton and the surface near the soliton predicted by the DFT calculation is quite complex. Further measurements, such as transmission electron microscopy for example, are required to directly observe the soliton and understand it electronic character.

## Conclusions

DFT simulations of the atomic and electron structure of (111) terminated Sb have been performed. The two possible surface models are considered, one containing van der Waals dangling bonds and one containing covalent dangling bonds at the surface. In the models the surfaces are infinite and the structure is defect free. Structural optimization of the Sb(111) surface containing dangling covalent bonds reveals the formation of a TS centered about 25 Å below the surface. The TS is well described within the SSH theoretical model. This TS represents a violation of the alteration between short covalent and long van der Waals bonds between Sb(111) atomic layers in the bulk. This strong deformation of the crystal lattice results in the formation of electronic states associated with the soliton. The TS interaction with the electrons of SS causes shift of the SS electronic bands. At the soliton core, the DOS at the Fermi level is observed to increase by an order of magnitude. The Fermi level DOS averaged over individual (111) planes demonstrates an oscillating dependence in the soliton region.

Monolayer steps have been observed by STM on the cleaved Sb(111) surface revealing that there exists different regions on the surface cleaved by van der Waals and covalent bonds. Based on the DFT calculation, we strongly suggest that areas with dangling covalent bonds upon cleavage reconstruct giving rise to the formation of the soliton below the surface. Experimentally the variation of monolayer step height as a function of the applied *U*_bias_—which is a result of DOS difference of the two surfaces—is 1.2–2.8 Å. This is comparable to the DFT calculated height variation between the fully relaxed vdW and CV surface models, the latter of which reconstructs to form the soliton. Furthermore, the DFT calculated (vdW and CV models of surface) and measured (either side of monolayer step) STS spectra are in agreement.

### Supplementary Information

Supplementary Information.

## Data Availability

The datasets used and/or analysed during the current study are available from the corresponding author on reasonable request.
