# Supplementary material for: The topological soliton in Peierls semimetal Sb
Source: Sci Rep. 2024 Jan 28;14:2331. doi: 10.1038/s41598-024-52411-x (PMC10822873; doi:10.1038/s41598-024-52411-x)
Supplement: Supplementary file 1 — Supplementary Information. [file 41598_2024_52411_MOESM1_ESM.docx]

**The topological soliton in Peierls semimetal Sb**

**SUPPLEMENTAL MATERIAL**

**A. Material**

To reduce the density of inhomogeneities in the Sb crystal, high purity Sb single crystals were used in our experiments. The high purity of the samples was confirmed by a high ratio of resistivity at 300K to resistivity at 4.2K – 2700. The electron mean free path at 4.2K was measured by electron focusing in a transverse magnetic field to be about 1mm. We have not detected any point defects in the STM images of the cleaved Sb(111) surfaces. On a few occasions we have observed traces of screw dislocations on the cleaved Sb(111) surfaces.

For the experiments, we used single-crystal Sb samples. (111) surfaces were obtained by cleavage the Sb single-crystals in UHV resulting in the formation of mirror planes on the surface. All stages of sample preparation were controlled *in situ* by LEED and XPS, and only samples without traces of contaminants in XPS spectra, and with LEED patterns characteristic for unreconstructed 1 × 1 surface of Sb(111), were studied by STM/STS, XPS and ARPES.

**B. STM/STS measurement details**

All experiments (LEED and STM/STS; LEED and XPS; LEED and ARPES; HRTEM) were carried out in independent UHV chambers with a base pressure in the range of 1 × 10^-9^ – 2 × 10^-10^ Torr or better. STM tips were cut from a PtIr single crystalline rod (around 0.28 mm in diameter). The bias voltage *U*_bias_ was applied to the sample with respect to the tip. No drift corrections have been applied to the STM images presented in this paper. All our STM experiments were performed on a large terraces far from the step edges.

**C. XPS measurement details**

Electronic structure and chemical composition were studied by XPS. High-resolution core level and valence band spectra were measured by a Kratos AXIS UltraDLD spectrometer (Kratos England), the photon energy was 1486.69 eV, Al *K_α_* mono, energy resolution at Ag 3d_5/2_ was about 0.48 eV. Pressure in the analytical chamber during spectral acquisition was less than 1 × 10^−9^ Torr. High-resolution spectra were acquired from 700 × 300 μm^2^ field of view with an analyzer pass energy of 20 eV . To show the sample composition spectra were acquired with a pass energy of 40 eV. The spectra were calibrated using the Ag 3d_5/2_ peak and were also referenced to the hydrocarbon C 1s peak at 284.7 eV.


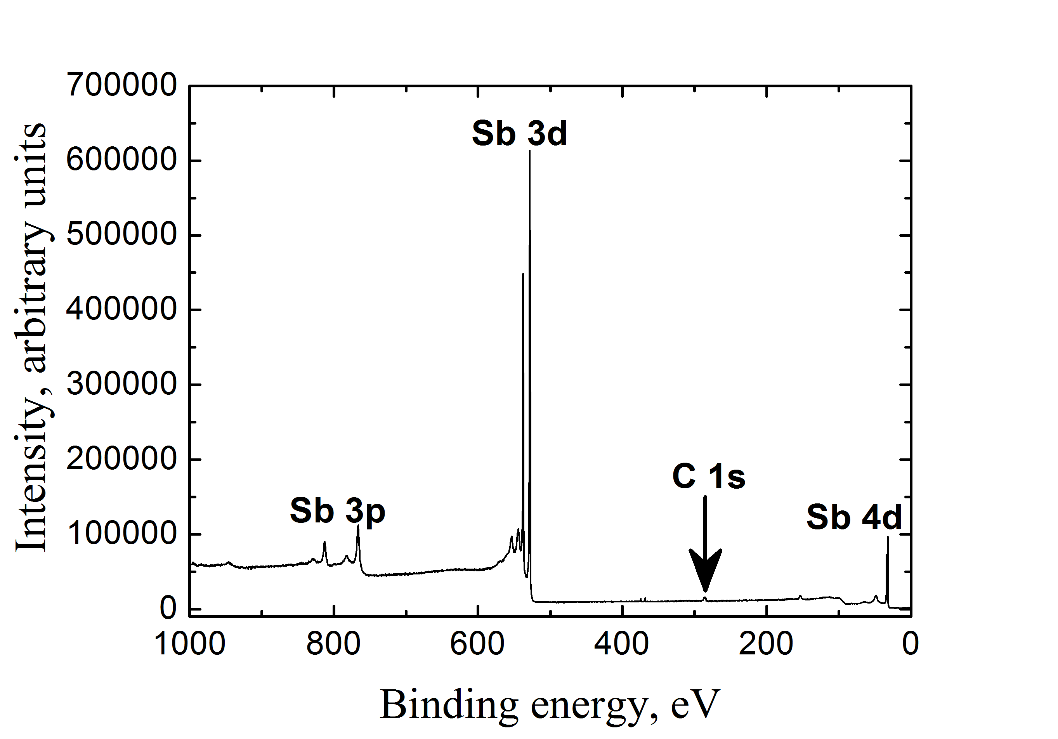


FIG. 1S. The XPS spectrum acquired on cleaved Sb(111) surface.

**D. ARPES measurement details**

The valence band structure was studied by ARPES at the Resource Center (RC) «Physical methods of surface investigation» of Research Park of Saint Petersburg State University using a photoelectron spectrometer equipped with a VG Scienta R4000 hemispherical electron energy analyzer. The spectra were acquired at room temperature and a photon energy of 40.8 eV (He IIα), with an angular resolution of 1° and a total energy resolution of 50 meV. The measurements were carried out using a PHOIBOS 150 electron energy analyzer with the 2D CCD detector system (SPECS GmbH). All spectra were acquired at RT.

**E. HR STEM measurement details**

Samples for the TEM investigations were prepared on VERSA HighVac dual beam microscope (FEI). The sample surface was protected by the carbon electron and ion induced deposition. The carbon deposition layer was done along the [001] trace of the cleavage, so that the plane with [110] zone axis would be aligned normal to the lamella surface. The sample preparation was carried out at 30 kV with a gallium source followed by the final polishing at 2 kV to improve the TEM lamella surface quality.

HAADF STEM images were collected on a Titan 80-300 probe corrected transmission electron microscope operated at 300 kV.

**F. Crystal structure of Sb9 and Sb21: DFT simulations HR STEM measurement details**


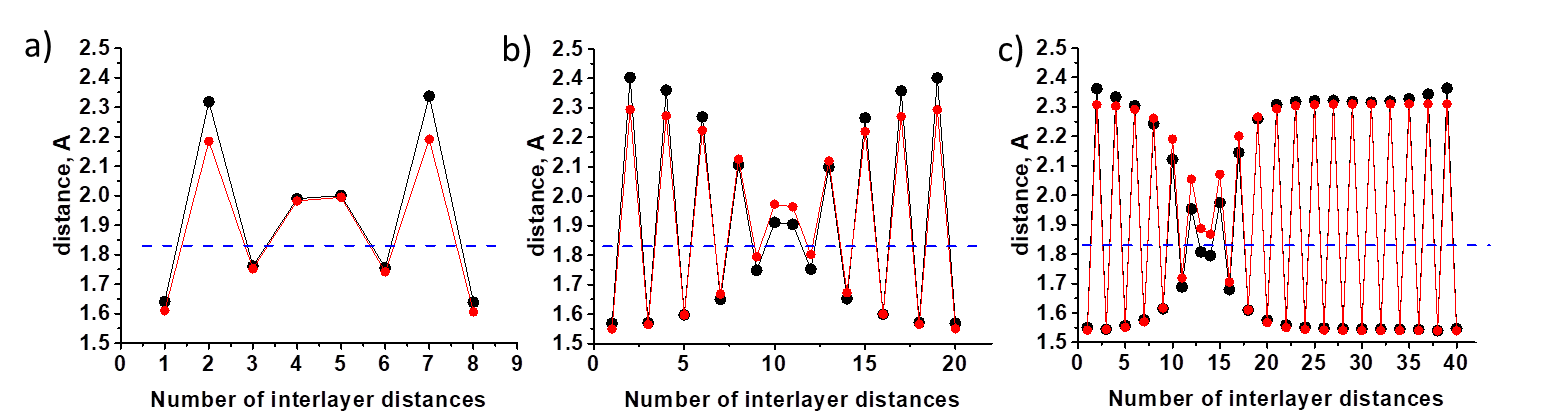


FIG. 2S. (a), (b) The dependence of the interplanar distance in the [111] direction on the number of the interlayer distance for Sb9 and Sb21 models, respectively.


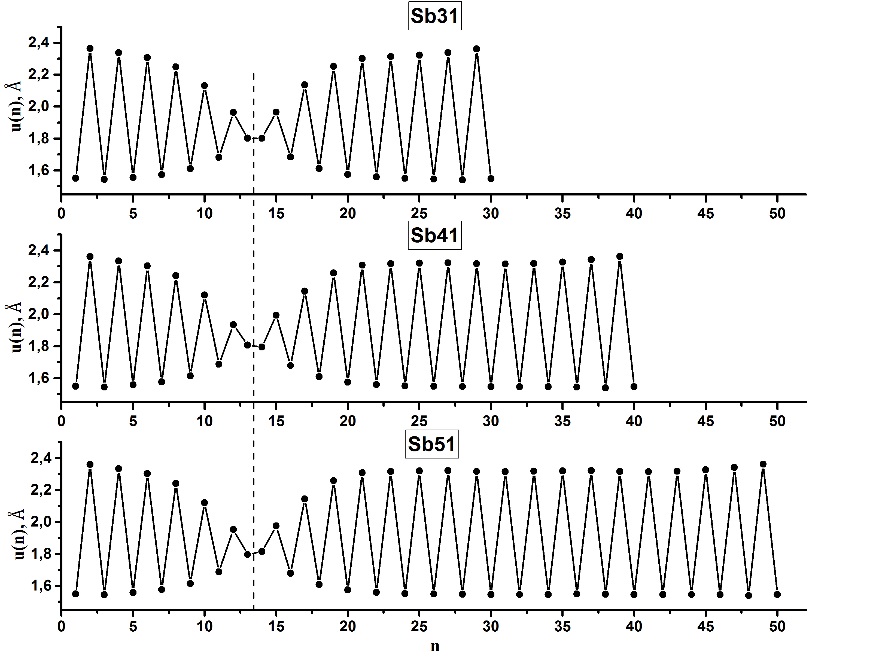


FIG. 3S. (a), (b) The dependence of the interplanar distance in the [111] direction on the number of the interlayer distance for Sb31, Sb41 and Sb51.

| TABLE 1S. Position and size of the soliton depending on the thickness of the structure under consideration. | | | |
| --- | --- | --- | --- |
| Thickness, atomic layers | *z*_0_, Å | Soliton size *w*, Å |  |
| 9 | 8.3 | 12.15 |  |
| 21 | 19.3 | 14.82 |  |
| 31 | 24.9 | 16.4 |  |
| 41 | 24.7 | 16.5 |  |

**G. Influence of step edges on the soliton position relative to the surface**

The soliton is predicted to form when covalent bonds are cleaved. The model considered in the text considers a surface where the entire surface is cleaved by the covalent bonds. In STM experiments, we observe finite (on the order of 100nm) region of the surface where covalent bonds have been cleaved. At the edge of these regions are step edges. We have considered the influence of the step edges on the soliton formation and position relative to the surface. The model, depicted in Fig. 4S, consists of a region where Van der Waals bonds are cleaved (vdW) and a region where Covalent bonds are cleaved (CV). These two regions are separated by a step edge at the surface. The black dashed line is where the soliton resides in the case where there is no step edges. In this model containing the step edge the soliton is localized closer to the surface. The deformation is indicated by the dashed red line. The deformation is pinned to the step edges and protrudes away from the surface at lateral distances further from the step edge.


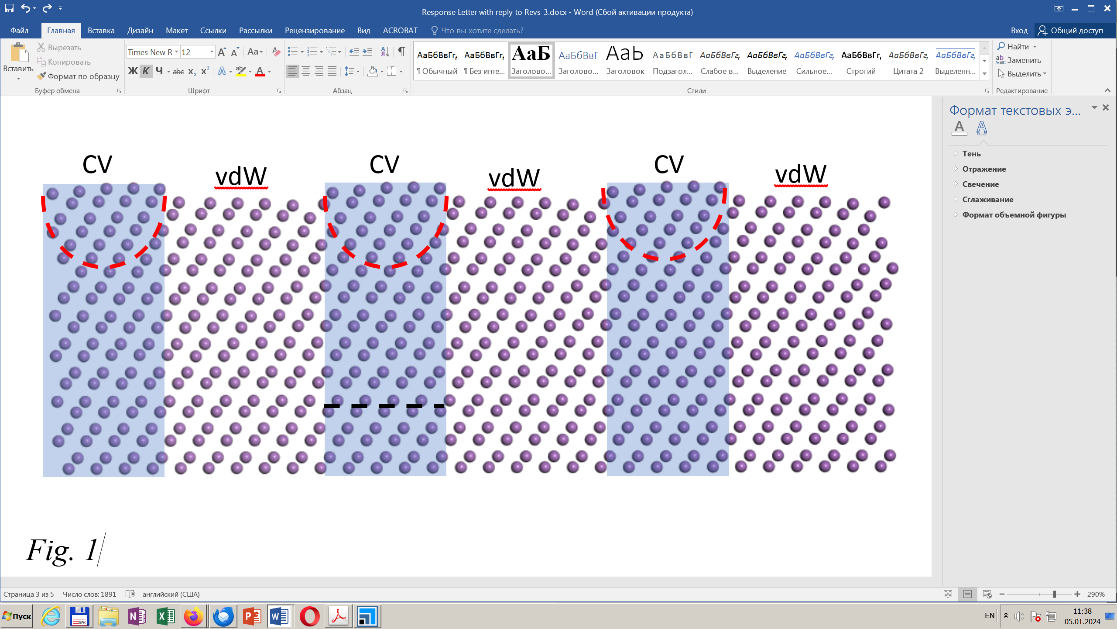

FIG. 4S Model consisting of a region where van der Waals bonds are cleaved (vdW) and a region where Covalent bonds are cleaved (CV). These two regions are separated by a step edge at the surface. The structural relaxation resulting from the cleavage of the covalent bonds (topological soliton) is illustrated by the red dashed line.

At the edges of the surface where covalent bonds are cleaved the step edges can influence the depth of the soliton. This effect will only be appreciable when the terrace width in on the nanometer scale. Typically, the terrace width on the cleaved surface is considerable larger. Therefore, at the realistic cleaved Sb(111) surface inhomogeneities associated with step edges are not important.

**H. Surface structure of Sb(111) after ion bombardment**

Ion bombardment of the cleaved Sb(111) surface was performed at room temperature by Ar^+^ ions. The Ar pressure was in a range of 5 × 10^-6^ – 4 × 10^-5^ mbar, etching area was about 2 × 2 mm^2^, and the beam energy of argon ions was *E_p_* = 4 keV.


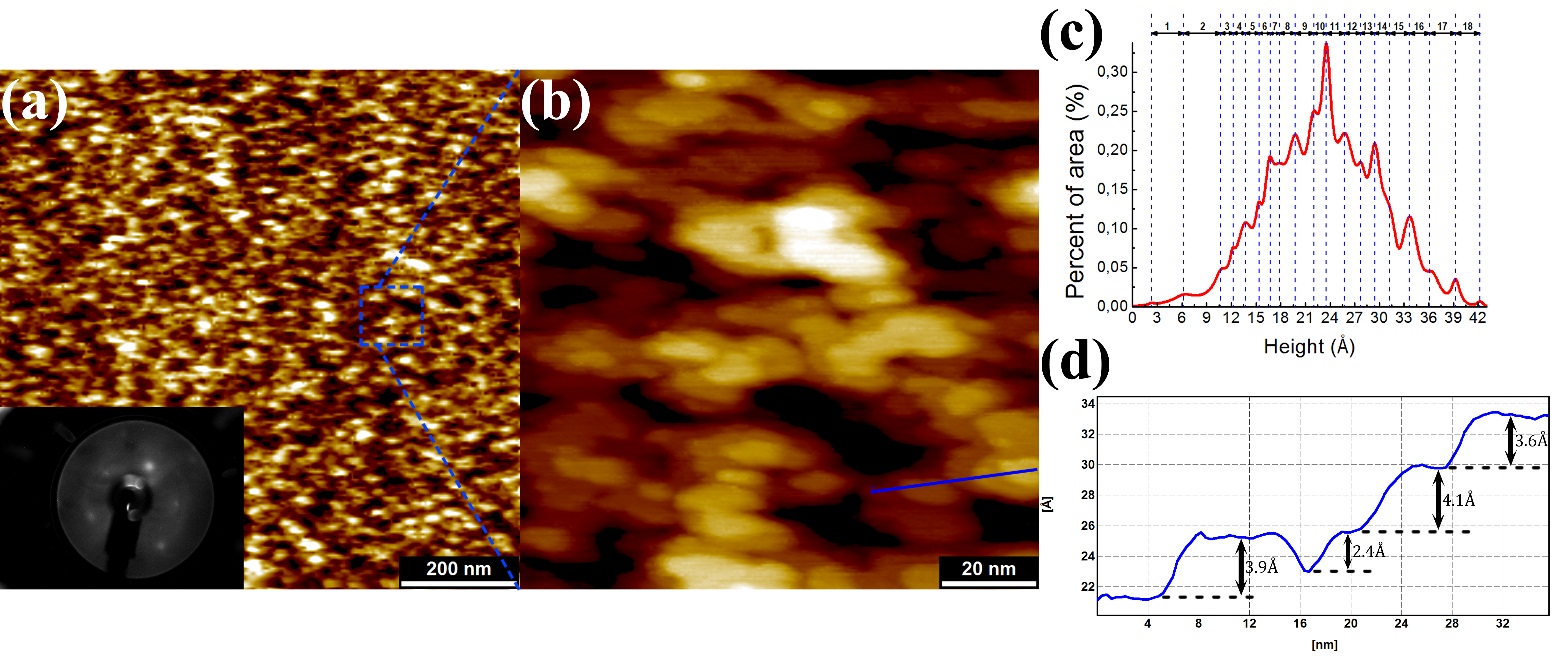


FIG 5S. (a) STM image of Sb(111) surface (900 × 900 nm^2^) after ion bombardment for 10 min measured with the Pt-Ir tip at *T* = 300 K, *U_bias_* = + 0.6 V, *I_tun_* = 200 pA. Insert – LEED pattern of Sb(111) surface after ion bombardment for 8 min. *E_p_* = 98.7 eV. The diffraction pattern was obtained on the electron spectrometer of Kratos AXIS Ultra DLD. (b) The same surface measured with higher magnification – 110 × 110 nm^2^ at *T* = 300 K, *U_bias_* = – 0.4 V, *I_tun_* = 300 pA. (c) Height distribution for the area of STM image (b) of etched Sb (111) surface. The distances between the peaks correspond to the interlayer spacing along [111] after a 10-minute ion bombardment: 1 – 3.9 Å, 2 – 4.5 Å, 3 – 1.5 Å, 4 – 1.5 Å, 5 – 1.6 Å, 6 – 1.3 Å, 7 – 1.1 Å, 8 – 1.9 Å, 9 – 2.3 Å, 10 – 1.5 Å, 11 – 2.2 Å, 12 – 1.9 Å, 13 – 1.7 Å, 14 – 1.8 Å, 15 – 2.4 Å, 16 – 2.5 Å, 17 – 3.2 Å, 18 – 2.9 Å. (d) The cross-section of the STM image (b) along blue line.


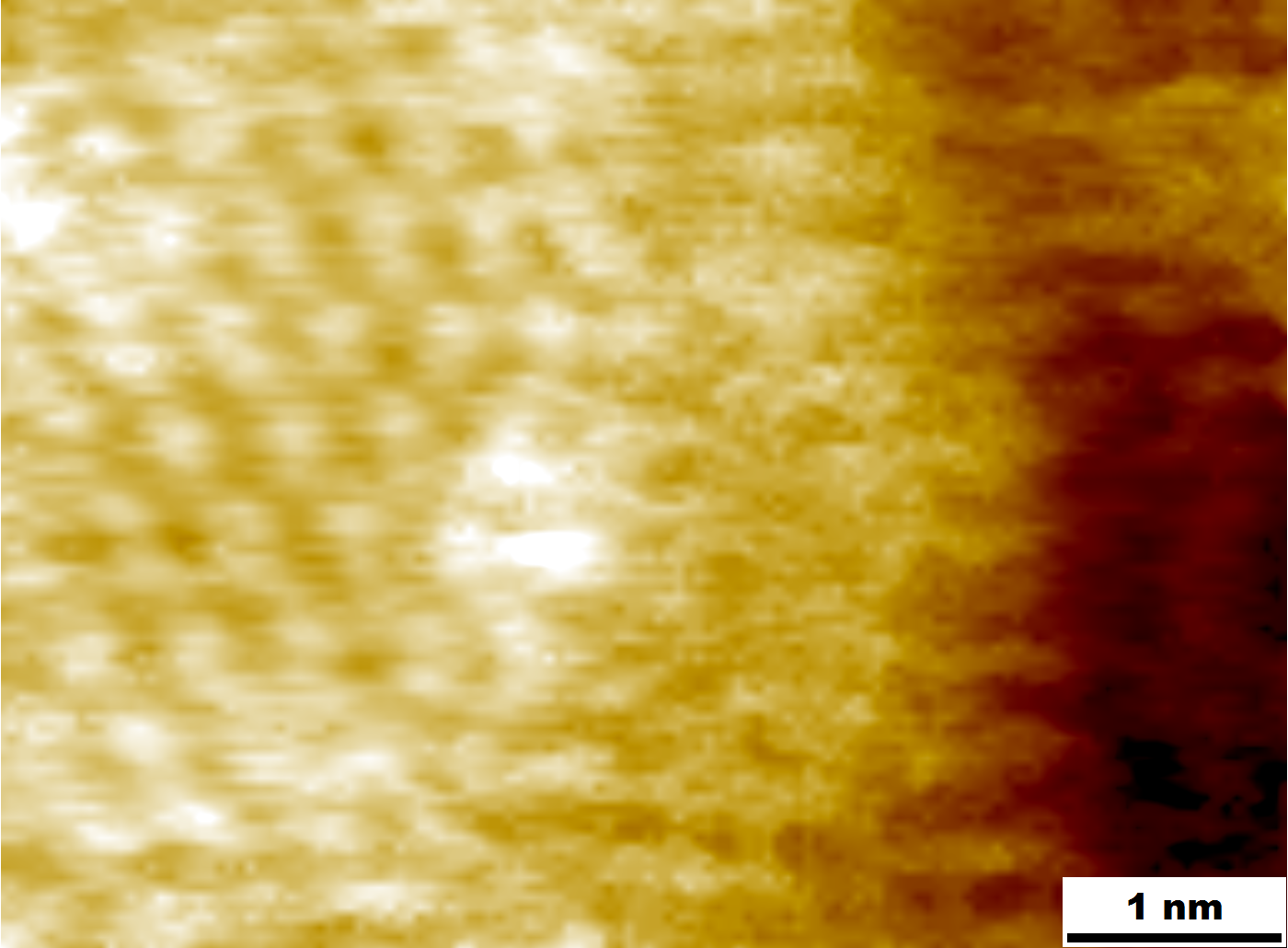

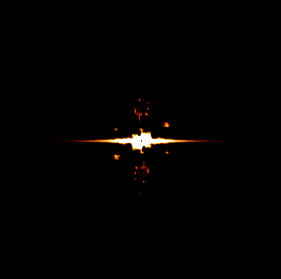


FIG 6S. (a) Atom-resolved STM image of Sb(111) surface (6 × 4.4186001 nm^2^) after ion bombardment for 30 min measured with the W[100] tip (coaxial Ar^+^ bombardment) at *T* = 300 K, *U_bias_* = + 0.01 V, *I_tun_* = 100 pA. Insert: The Fast Fourier transform of this STM image.


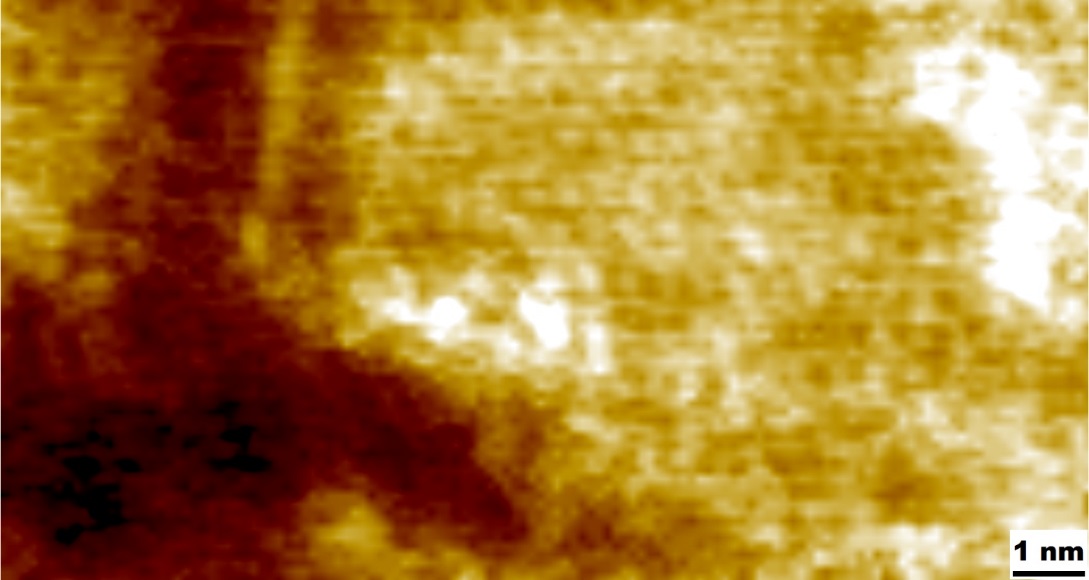

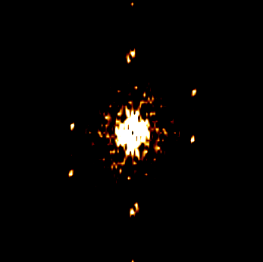


FIG 7S. (a) Atom-resolved STM image of Sb(111) surface (15 × 8.0215998 nm^2^) after ion bombardment for 30 min measured with the W[100] tip (coaxial Ar^+^ bombardment) at *T* = 300 K, *U_bias_* = + 0.01 V, *I_tun_* = 100 pA. Insert: The Fast Fourier transform of area where is resolved of the Sb atoms.

**J. Calculation of the surface formation energies**

*E_vdW_, E_S_* and *E_CV_* were determined from DFT simulations. In order to calculate *E_vdW_ and E_CV_* the total energy of the following structures were calculated:


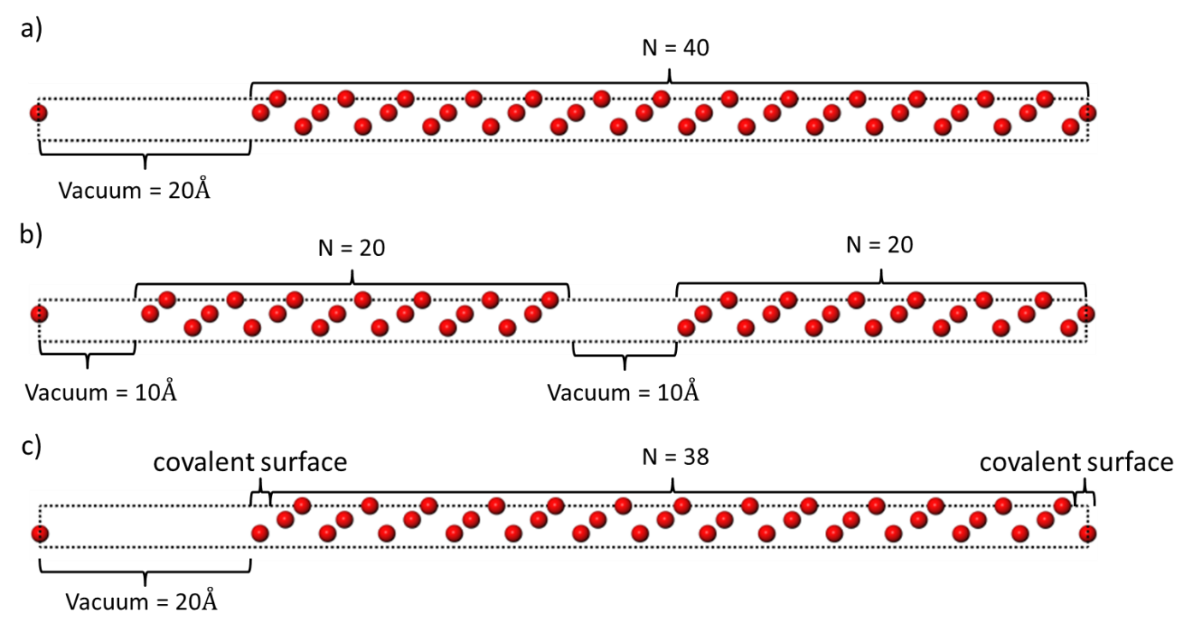


FIG 8S.

*E_a_=2E_vdW_+38E_bulk_*,
*E_b_=4E_vdW_+36E_bulk_*,
*E_c_=2E_CV_+38E_bulk_*
where *E_bulk_* is the atomic layer energy in the bulk of the slab. Solving these equation we obtain the values of *E_vdW_* and *E_CV._ E_S_*=0.78eV is the difference in energy between the relaxed and unrelaxed 41Sb structures.
